# Make It, Take It, or Leave It: Heme Metabolism of Parasites

**DOI:** 10.1371/journal.ppat.1003088

**Published:** 2013-01-17

**Authors:** Luděk Kořený, Miroslav Oborník, Julius Lukeš

**Affiliations:** 1 Biology Centre, Institute of Parasitology, Czech Academy of Sciences, České Budějovice (Budweis), Czech Republic; 2 Department of Pathology, University of Cambridge, Cambridge, United Kingdom; 3 Faculty of Science, University of South Bohemia, České Budějovice (Budweis), Czech Republic; 4 Institute of Microbiology, Czech Academy of Sciences, Třeboň, Czech Republic; University of Wisconsin Medical School, United States of America

Heme and other tetrapyrroles, often called “the colors of life”, belong to the most important molecules of almost all extant organisms. They are synthesized by a common multistep pathway that is highly conserved throughout the tree of life [1]. One of the tetrapyrrole products is chlorophyll, the green pigment of plants and other phototrophs, which captures the energy of the sun. Vitamin B12, the most complex tetrapyrrole, is involved in DNA synthesis and energy metabolism [2]. The major product of tetrapyrrole biosynthesis in non-photosynthetic organisms is heme, an iron-coordinated porphyrin with the capacity to transfer electrons and bind diatomic gases. Here we summarize the current understanding of different aspects of heme metabolism in parasitic eukaryotes, including the synthesis and uptake of heme and its detoxification. A differential need for heme in distinct parasitic groups and the suitability of heme metabolism as a drug target for treating parasite-borne diseases are also discussed. First, however, let us review the functions of heme in various cellular processes.

## What Are the Functions of Heme and How Does It Relate to Parasites?

Probably the best known function of heme is the transport of oxygen as part of the hemoglobin molecule, which gives the red color to blood. Unicellular organisms do not need to transport oxygen throughout tissues, but may still use globins or other hemoproteins to sense diatomic gases and to avoid oxidative or nitrosative stress [3]. This might have a major impact for the life cycles of parasites, as the immune response of their hosts is often based on the production of reactive oxygen (ROS) or nitrogen species. Furthermore, many parasites adapted to anaerobic environments, with oxygen being toxic to them. Some anaerobic parasites possess flavohemoglobin or other hemoproteins, which have either the capacity to consume nitric oxide and oxygen or serve as signal transducers upon their binding [4] (Table 1).

**Table 1:** Heme proteins of selected parasitic groups.

		Aerobes							Anaerobes or Microaerophils							
Processes/Heme Proteins		*Crithidia*	*Leishmania*	*T. brucei*	*T. cruzi*	*Phytomonas*	*Plasmodium*	*Toxoplasma*	*Cryptosporidium*	*Trichomonas*	*Giardia*	*Entamoeba*	*Encephalitozoon*	*Antonospora*	*Nematocystis*	*Blastocystis*
Respiration	Cytochrome subunit of succinate dehydrogenase	✓	✓	✓	✓	✓	✓	✓								✓
	Cytochrome *c*	✓	✓	✓	✓		✓	✓								
	Cytochrome *b* subunit of complex III	✓	✓	✓	✓		✓	✓								
	Cytochrome *c1* subunit of complex III	✓	✓	✓	✓		✓	✓								
	Cytochrome *a* subunit of complex IV	✓	✓	✓	✓		✓	✓								
Oxidative/nitrosative stress defense	Heme peroxidases	✓	✓	✓	✓											
	Catalase	✓						✓						✓		
	Flavohemoglobin										✓					
Signal transduction	Adenylate/guanylate cyclase (globin domain)	✓	✓													
	Adenylate/guanylate cyclase (PAS domain)									✓						
	Protein kinases (PAS domain)	✓	✓	✓	✓	✓			✓							
	Phosphoglycerate kinase (PAS domain)	✓	✓		✓	✓										
	DNA-binding HLH protein (PAS domain)															✓
	Heme-binding SOUL protein								✓							
Sterol synthesis	Lanosterol 14α-demethylase (cytochrome P450)	✓	✓	✓	✓	✓										
	C-22 sterol desaturase (cytochrome P450)	✓	✓		✓											
Other redox reactions	Ferric reductases	✓	✓	✓	✓											
	Cyt *b*_5_ domains of fatty acid desaturases	✓	✓	✓	✓											
	Cyt *b*_5_ domain of fatty acid hydroxylase							✓								
	Cyt *b*_5_ domain of sulfite oxidase							✓								
	Cyt *b*_5_ domain of nitrate reductase	✓	✓	✓	✓											
	Other cytochromes *b*_5_	✓	✓	✓	✓	✓	✓	✓	✓	✓	✓		✓	✓	✓	
	Other cytochromes P450	✓	✓	✓	✓			✓								

The fact that heme iron can exist in either a reduced or oxidized state relates to most functions of heme participating in electron-transport systems and redox reactions within the cell. All organisms obtaining energy via oxidative phosphorylation need large amounts of heme for the cytochromes of the mitochondrial respiratory chain. Cytochromes of the *b*_5_ or P450 families transfer electrons in reactions such as desaturation of fatty acids, detoxification of various drugs, or biosynthesis of sterols [5]. As a component of peroxidases and catalases, heme is able to consume hydrogen peroxide and therefore has a great impact on the ROS balance. Although these enzymes are likely crucial for the protection of parasites from the oxidative burst of their hosts, their activities may also have a negative impact on their virulence, since the host's ROS production serves as a signal during the parasites' life cycles [6]. Due to the important roles of heme in the elementary cellular processes of most organisms, heme metabolism is considered a promising target for new anti-parasitic drugs [7], [8]. Recently, biosynthesis of heme has been suggested as a promising target for treating malaria, since its causative agent *Plasmodium* uses a unique route of heme synthesis [9]. To demonstrate how this pathway evolved, we first describe how heme biosynthesis looks in other eukaryotes.

## How Is Heme Synthesized and How Do Parasites Obtain It?

**Figure 1:**
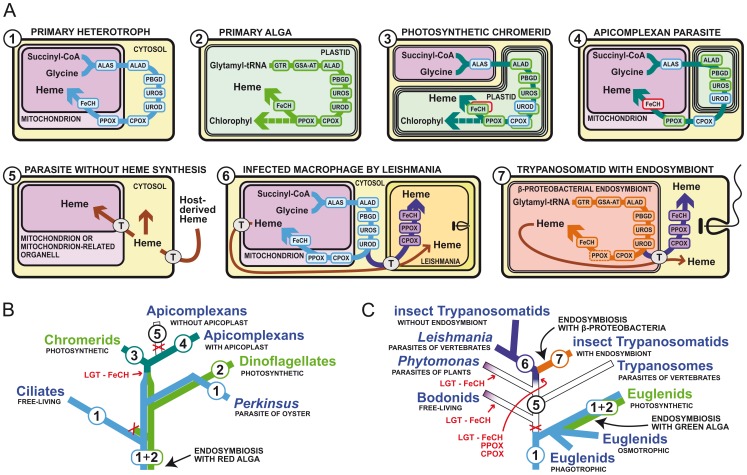
Different ways of heme acquisition and their distributions within alveolates and kinetoplastids. (A) Intracellular localizations of heme synthesis enzymes and heme trafficking in various parasites. The colors and numbers of the phylogenetic lineages in (B) and (C) correspond to the colors of the pathways and numbers in the schematics in (A). The blue line (no. 1) represents the original pathway of the primary heterotroph and is similar to the well characterized C4 pathways of animals and fungi. The green line (no. 2) represents the plastidial C5 pathway of the endosymbiotic alga and is present in most phototrophs. In the ancestral lineages of alveolates, both of these pathways co-existed as in extant photosynthetic euglenids. The lines no. 3 and 4 represent the hybrid pathways of photosynthetic chromerids and apicomplexan parasites, which resulted from the combination of the original pathway of the host (no. 1) and the plastidial pathway from the algal endosymbiont (no. 2). FeCH in a red box was acquired via lateral gene transfer (LGT) from a γ-proteobacterium. The purple line (no. 6) represents the partial pathway of the trypanosomatid flagellates, which consists of the last three steps encoded by enzymes of γ-proteobacterial origin. The orange line (no. 7) is the heme synthesis pathway of the β-proteobacterial endosymbionts of one group of trypanosomatids. T stands for porphyrin transporters. (B) Evolution of the heme biosynthesis in Alveolata leading to the unique pathway of apicomplexan parasites. (C) Evolution of the heme synthesis in Euglenozoa leading to the loss of the pathway in ancestral kinetoplastids and its partial rescue by bacterial genes for the last three steps and full rescue by bacterial endosymbionts in trypanosomatids. The abbreviated enzyme names are: ALAS, ALA-synthase; GTR, glutamyl-tRNA reductase; GSA-AT, glutamate 1-semialdehyde aminotransferase; ALAD, ALA-dehydratase; PBGD, porphobilinogen deaminase; UROS, uroporphyrinogen synthase; UROD, uroporphyrinogen decarboxylase; CPOX, coproporphyrinogen oxidase; PPOX, protoporphyrinogen oxidase; FeCH, ferrochelatase.

**Figure 2:**
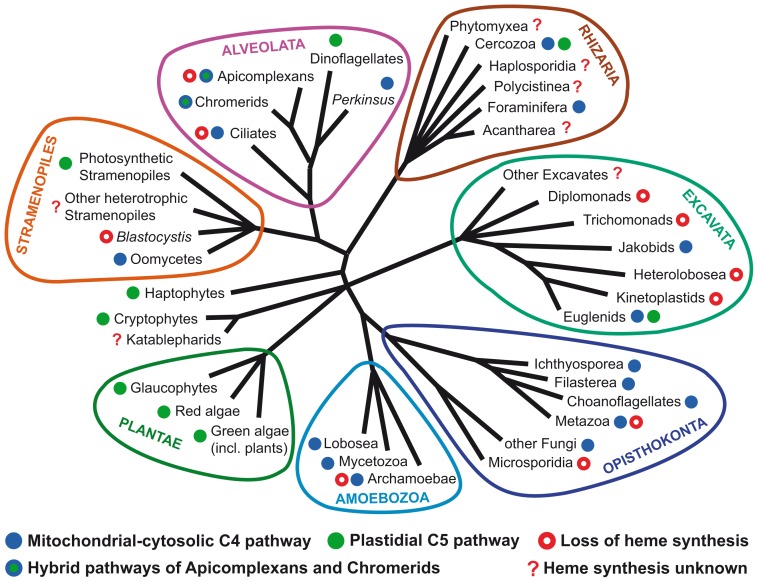
Distribution of different types of heme synthesis and independent losses of heme biosynthetic pathways in eukaryotes.

Most organisms are able to synthesize their own heme in a pathway in which the first committed precursor, δ-aminolevulinic acid (ALA), is converted to heme by seven universally conserved enzymatic steps. Archaea and most bacteria synthesize ALA from glutamate (C5 pathway), while α-proteobacteria do it by condensation of succinyl-CoA with glycine (C4 pathway) [1] (Figure 1A). Heme biosynthesis in eukaryotes has largely been influenced by endosymbioses, resulting in the evolution of mitochondria and plastids. Primary heterotrophic eukaryotes clearly combined the original synthetic pathway of the pre-eukaryotic host with that of the α-proteobacterial predecessor of mitochondrion, resulting in an evolutionary mosaic pathway [10]. This pathway spans both the cytosol and the mitochondrion, where heme is mostly needed for respiratory cytochromes (Figure 1A). Photosynthetic eukaryotes acquired another tetrapyrrole synthesis pathway from the engulfed cyanobacterium transformed to a primary plastid, or from a eukaryotic alga already possessing a plastid evolving into a secondary plastid. The plastidial pathway serves mainly for the synthesis of chlorophyll, which is required in much greater amounts than heme. Uniquely, both the mitochondrion-cytosolic and the plastidial pathways co-exist in *Euglena gracilis*, which acquired its plastid through a secondary endosymbiosis relatively recently (Figures 1A, 1C, and 2) [11]. In most other phototrophs, the plastidial pathway took over tetrapyrrole synthesis and the original pathway of the host cell disappeared [10]–[12].

Apicomplexan parasites possess a most unusual type of heme synthesis, as it spans three cellular compartments and uses enzymes of various origins (Figure 1A). The photosynthetic ancestry of apicomplexans is well known. These parasites retain a relic secondary plastid, called the apicoplast, which is still required for the syntheses of isoprenoids, fatty acids, and heme [13]. Recent characterization of the heme synthesis pathway of apicomplexans, as well as that of chromerids, their photosynthetic relatives [10] (Figure 1B), revealed that in both lineages the mitochondrion-cytosolic pathway of the host and the plastidial pathway of the endosymbiont contributed to their unusual tetrapyrrole pathways (Figures 1A and 1C). In *Chromera velia*, the synthesis starts with the heterotrophic C4 pathway in the mitochondrion, but both heme and chlorophyll are produced in the photosynthetic plastid. However, having no need for these products in their non-photosynthetic plastids, apicomplexans redirected the last steps of the synthesis to the cytosol and mitochondrion, where heme is produced mainly for respiration [10] (Figure 1C).

In contrast to the apicomplexans, many other parasitic groups have lost the capacity to synthesize heme (Figure 2). The colonization of anaerobic environments, which makes heme synthesis impossible because of the two oxygen-requiring steps in the pathway, was probably one of the reasons for this loss. Although some bacteria are known to possess oxygen-independent analogs of these enzymes [1], so far they have never been found together in any eukaryote (unpublished data). Moreover, the anaerobes need only a little heme for the few heme proteins they encode in their genome (Table 1), and it is perhaps easier to obtain it from their hosts. Easy access to heme from external sources was likely the reason why even some aerobes, which need heme for mitochondrial respiration and oxygen metabolism in general, lost the heme synthesis pathway. The accumulation of either heme or its precursors leads to the production of ROS and cell damage [14], and it is therefore advantageous to get rid of heme synthesis whenever possible. These organisms must have evolved mechanisms of heme acquisition, such as heme binding proteins and porphyrin membrane transporters. At present very little is known about such transporters [15]–[17], while the mechanism of heme import into the mitochondria of heme auxotrophs remains unknown.

The evolution of heme acquisition within the group Kinetoplastea, which includes serious human parasites of the genera *Trypanosoma* and *Leishmania* (Figures 1A and 1C), is very interesting. These protists likely lost the pathway before they became parasites, since even the basal free-living bodonids lack a heme synthesis pathway and acquire heme from the bacteria they feed on [18], [19]. Trypanosomes are heme auxotrophs, taking up heme from the host, while *Leishmania* and insect trypanosomatids may use later precursors of heme synthesis as well, as they encode enzymes for the last three steps of the pathway. The genes involved have been acquired from γ-proteobacteria by lateral gene transfer (LGT) [18]. In addition, some insect trypanosomatids harbor bacterial endosymbionts that supply them with heme or its precursors, thus resembling organelles [18], [19]. The evolution of heme synthesis in kinetoplastids thus went in the opposite direction than in most parasitic lineages. Although in kinetoplastids many metabolic pathways have been reduced or lost due to their parasitic lifestyle, heme synthesis has been rescued from complete absence in their ancestors to a fully functional biosynthetic pathway in the endosymbiont-containing insect trypanosomatids.

## Do All Parasites Require Heme?

The requirement for heme usually correlates with the simplification of a parasites' metabolism and with the presence of oxygen in their environment. As most heme functions relate to oxygen in one way or another, anaerobic species possess only a very few hemoproteins (Table 1), mostly involved in sensing diatomic gases and avoiding oxidative and nitrosative stresses. Microsporidia, intracellular parasites related to fungi, greatly simplified their metabolism. They possess the smallest known eukaryotic genomes and obtain most nutrients from the host cells [20]. *Encephalitozoon cuniculi* seems to encode only one hemoprotein, a member of the cytochrome *b*_5_ family of unknown function, while *Antonospora lacustae* also possesses catalase, which is active in the dormant stage [21] (Table 1). Although no heme protein is listed in Table 1 for *Entamoeba*, this amoeboid human parasite secretes two proteins that bind heme in vitro. However, they were suggested to scavenge for heme not for heme itself but because of the requirement for iron [22]. Thus the possibility remains that this anaerobe is totally devoid of heme proteins.

The aforementioned parasites are biotrophs that cannot be cultivated in chemically defined media, meaning that their dependence on heme cannot be rigorously tested. The only known eukaryote able to prosper without heme is the kinetoplastid parasite of plants *Phytomonas* [23]. Surprisingly, this aerobe retains several cellular processes normally involving heme. *Phytomonas* is still able to desaturate fatty acids, likely using an alternative electron donor. The heme-requiring respiratory complexes are bypassed by an alternative oxidase, and sterol biosynthesis can be stopped before the heme-requiring demethylation step. Ergosterol, which is normally synthesized in the presence of heme, can be substituted with its precursor, lanosterol, without any effect on the in vitro growth of this flagellate [23]. Although the use of heme likely provides some advantage at least in part of its life cycle, this organism is living proof that even under aerobic conditions all heme functions can potentially be lost or bypassed when so much energy is provided by glycolysis that oxidative phosphorylation-derived ATP is not required.

## How Do Parasites Cope with Heme Toxicity?

While useful for many cellular processes, free heme induces the production of ROS and causes lipid peroxidation, resulting in membrane injury and cell apoptosis [14]. The large quantities of heme released during hemoglobin digestion are a particular challenge faced by parasites dwelling in or feeding on blood. These parasites have evolved various detoxification mechanisms, including the breakdown of heme into molecular iron and less reactive intermediates, the containment of heme by a physical barrier, and the conversion of heme into an inert crystalline structure [14]. The formation of hemozoin, a single unit heme polymer, by *Plasmodium* is well known, but this mechanism has also been described in the blood-feeding human parasitic worms *Schistosoma* spp. and in blood-sucking triatomine bugs [8]. A slightly different strategy is employed by ticks, which accumulate heme as a non-crystalline aggregate in a specialized organelle called hemosome [14]. The ability to break heme is still questionable for many parasites. It is normally executed by heme oxygenase (HO), which leads to the production of biliverdin that is further metabolized to bilirubin, a non-toxic water-soluble pigment. Although HO has recently been identified in *Plasmodium* [24], its presence or absence remains unknown for many parasites, including kinetoplastids. *Leishmania* seem to be able to break down heme to be used as a source of iron [25], while the heme and iron acquisition pathways are likely independent in other parasites [26].

## Is Heme Metabolism a Good Drug Target?

Heme synthesis is present only in certain parasites and was regarded as a potential drug target mainly for *Plasmodium* and other apicomplexans, where it partially localizes to the apicoplast. However, it has been shown recently that of the three plastid-localized pathways only isoprenoid synthesis is indispensable for the erythrocytic stage, since addition of isopentenyl pyrophosphate chemically rescues an elimination of the apicoplast in *Plasmodium* [27]. Heme that is still required in this life stage can potentially be acquired from the host blood or synthesized using host-derived enzymes [28]. Other *Plasmodium* life stages are likely dependent on their own biosynthetic pathway. In contrast, the anaerobic *Cryptosporidium*, and likely some gregarines, have lost the capacity to synthesize heme, and a detectable apicoplast is absent from their cells as well [10], [28]. This indicates that it is the need to synthesize heme which prevents this organelle from being lost. Nevertheless, we argue that *Plasmodium* heme synthesis is not a suitable drug target. On the other hand, the pathway of heme detoxification proved to be an excellent therapeutic target for malaria and other parasites. The antimalarial quinoline, for example, limits intra-erythrocytic parasite growth by inhibiting hemozoin formation and is also effective against *Schistosoma* [8], [28].

Although no kinetoplastid can synthesize heme using early precursors, *Leishmania* spp. encode the last three steps of the synthesis, while the first five enzymes of the pathway are absent [18], [29] (Figure 1C). It remains to be tested whether these enzymes are really used for the conversion of the host-derived coproporphyrinogen into heme. If so, protoporphyrinogen oxidase would be a promising drug target, as *Leishmania* spp. and some other trypanosomatids are the only known eukaryotes that possess the bacterial enzyme, which differs from the eukaryotic analog not only in its sequence, but also in its mechanism of action. This step of heme synthesis has been successfully targeted in plants by various herbicides causing the accumulation of porphyrins, which upon exposure to light trigger oxidative damage [30]. The same principle is also used in cancer treatment by photodynamic therapy.

The fact that it is necessary for heme auxotrophs to import heme via specialized transporters provides other potential drug targets, as exemplified by the essentiality of the recently identified porphyrin importer in *Leishmania* [16]. A specific haptoglobin-hemoglobin receptor that mediates heme uptake has been described in the bloodstream stage of *Trypanosoma brucei* [15]. However, contrary to *Leishmania*, which requires substantial amounts of heme for mitochondrial respiration, this life stage of *T. brucei* relies on glycolysis as the sole energy source. The inaccessibility of heme in vivo resulted in decreased growth caused by higher sensitivity to oxidative stress, likely due to a change in the membrane lipid composition. Inhibition of the key enzymes of macrophage oxidative burst rescued the parasite growth [15], indicating that heme may not be required for the viability of this life stage of *T. brucei*, the metabolism of which is similar to the related *Phytomonas*. This would make the pathway of heme uptake in *T. brucei* a less attractive drug target, similar to the heme synthesis pathway of the erythrocytic stage of *Plasmodium*.
